# Wavefront Control with Nanohole Array-Based Out-of-Plane Metasurfaces

**DOI:** 10.1021/acsanm.1c01178

**Published:** 2021-08-02

**Authors:** Mohsin Habib, Ibrahim Issah, Daria Briukhanova, Alireza R. Rashed, Humeyra Caglayan

**Affiliations:** Faculty of Engineering and Natural Science, Photonics, Tampere University, 33720 Tampere, Finland

**Keywords:** metasurfaces, rolled-up tubes, wavefront control, nanoholes

## Abstract

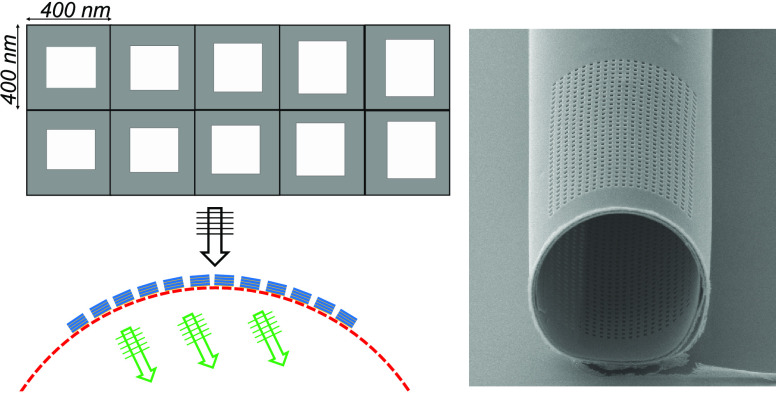

Planar metasurfaces provide exceptional wavefront manipulation at the subwavelength scale by controlling the phase of the light. Here, we introduce an out-of-plane nanohole-based metasurface design with the implementation of a unique self-rolling technique. The photoresist-based technique enables the fabrication of the metasurface formed by nanohole arrays on gold (Au) and silicon dioxide (SiO_2_) rolled-up microtubes. The curved nature of the tube allows the fabrication of an out-of-plane metasurface that can effectively control the wavefront compared to the common planar counterparts. This effect is verified by the spectral measurements of the fabricated samples. In addition, we analytically calculated the dispersion relation to identify the resonance wavelength of the structure and numerically calculate the phase of the transmitted light through the holes with different sizes. Our work forms the basis for the unique platform to introduce a new feature to the metasurfaces, which may find many applications from stacked metasurface layers to optical trapping particles inside the tube.

## Introduction

In recent years, metamaterials with unusual electromagnetic properties have provided extensive control on the response of electromagnetic waves with the arrangement of subwavelength antennas.^1,2^ Particularly, the two-dimensional counterparts of metamaterials (metasurfaces) have been extensively studied to achieve applications such as metalenses,^3−5^ beam steering devices,^6^ color filters,^7^ visual gas sensing,^8^ holography,^9^ and optical trapping^10^ devices. Different levels of manipulation and efficiency were obtained in these applications, which are provided by the arrangement of different antennas such as V-shaped antennas,^11^ elliptical,^12^ square nanoposts,^13^ and perforated nanovoids or nanoholes.^14,15^ Especially, the nanohole arrays contribute to the flexible metasurface platforms with different applications due to their top-down approach for incorporating various materials.^16^

Notably, when these nanohole arrays are obtained on the structures comprising stacked metal-dielectric layers, the spectral response is much richer as the additionally supported surface plasmon polaritons (SPP) modes arise. Not only single-interface (external) SPP^17^ but also a gap (internal) SPP^18,19^ are supported in these nanohole arrangements introduced on a thin metal layer or metal/dielectric stacks. Over the last several years, these nanohole arrays delivered interesting features and applications from extraordinary transmission^20^ to enhanced biosensing^21^ and realization of the negative refractive index.^22−24^ Lately, Matsui et al. brought metal-dielectric hole arrays to the metasurface applications using different shapes to control the phase of the transmitted light.^25^ Additionally, an inclined wavefront for beam steering in the near-infrared range has been achieved using a gradual change in the hole size.^26^ These inverted metasurfaces in contrast to the regular ones lead to a significantly higher signal-to-noise ratio and efficient focusing of the incident light.^15^ However, the fabrication of such structures is typically based on subsequential layer deposition of metal and dielectric, which require precise control on the deposition of each layer. For research applications, such an approach is not only time-consuming but also limited by the uniformity and reproducibility of each layer, given the involvement of multiple steps of deposition. Yet, a unique self-rolling mechanism of multilayer metal and dielectric/semiconductor materials with fewer deposition steps can provide an excellent solution to these challenges. The strain-induced self-rolling method, known as the thin-film self-rolling technique for three-dimensional (3D) rolled-up tubes (RUTs), has been used in different fields after it was introduced in semiconductor bilayers grown by molecular beam epitaxy (MBE),^27^ including electronic,^28^ magnetoelectronic devices,^29^ single-cell culture study,^30^ and biological sensing.^31−33^ Although applying a self-rolling method to fold more complex structures opens new ways to produce 3D photonic micro-objects with novel designs and optical properties, it has not been adopted yet into metasurfaces.

This work introduces another degree of control over the design of planar metasurfaces by providing an out-of-plane contribution to wavefront modification. We utilized the curvature of strain-driven self-rolling three-dimensional RUTs formed of gold (Au) and silicon dioxide (SiO_2_) layers to obtain a unique metasurface with rectangular nanohole arrays. Nanoholes were fabricated on the multilayer metal/dielectric walls of the RUTs with different sizes to introduce a gradual shift to the phase of the transmitted light. While enriched dispersion provides the coupling to hybrid plasmon modes, the metasurfaces on the RUTS wall provide additional degrees of freedom to modify the dispersion of the planar structures. The circular curvature of RUTs provides an extra phase difference that leads to an additional wavefront control mechanism compared to the planar metasurface designs. Our approach of using RUTs does not only reduces the fabrication time and the related challenges but also grants a powerful and unique wavefront control and dispersion engineering platform. The integration of self-rolling systems with the advanced metasurfaces enables various unique optical functionalities from stacked nonlinear^34,35^ metasurface layers to optical trapping of particles inside the tube.

## Metasurface Design

To facilitate the properties of the modes in nanohole-based metasurface design, we implemented the dispersion relation of the stacked metal-dielectric film and illustrate the different plasmon mode excitations using the Helmholtz equations (see the Supporting Information for details). Although the proposed formulation is an approximation of the metasurface design, it can be extended to the general case of multiple layers with random permittivities. This formulation is used to elucidate the properties of SPPs, the propagating modes on the surface, and the inside of the stacked hole metal-dielectric layers. Predominately, localized surface plasmon excitation in metamaterials is relevant due to its distinguishing feature of confining an optical field in the subwavelength regime and guiding the optical field to a relatively long distance. These external and gap plasmon modes, excited by the metamaterials with holes, have been of interest and used to understand the acquired resonances in the reflectance spectra.^36^

**Figure 1:**
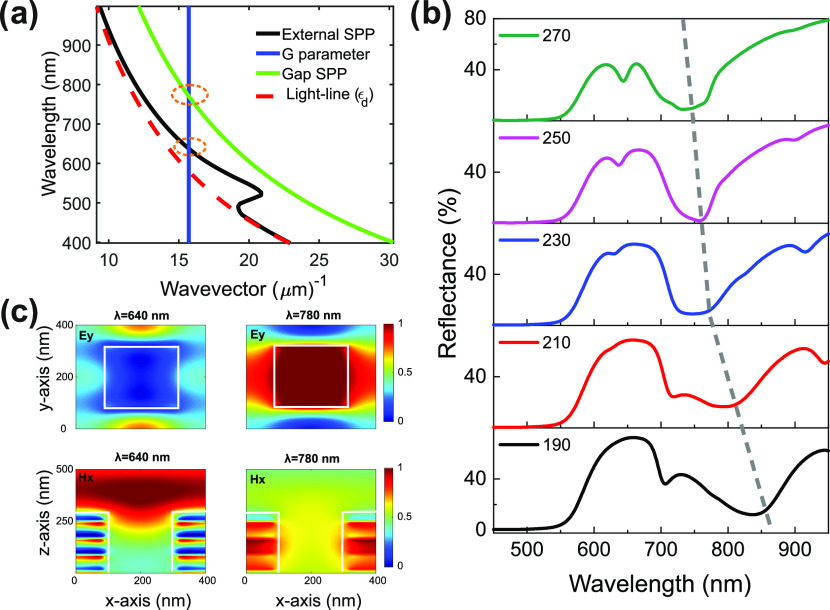
(a) Dispersion curves of the external and gap SPP modes excited by metamaterials milled with holes. The light-line ϵ_d_ shows the dispersion relation of the dielectric medium and the G parameter corresponds to the reciprocal lattice vector of the stacked holes. The two orange circular insets illustrate the relation of SPPs and the conservation of momentum as a function of the lattice periodicity. (b) Reflectance curves of five different hole sizes. The gray line shows the blue shift in the reflectance spectra as a function of different hole sizes. (c) *y* component of electric field (*Ey*) in the *x*−*y* plane from the top at 640 and 780 nm are presented in top panels. Similarly, a cross section view of the *x* component of magnetic field (H_*x*_) at 640 and 780 nm are presented in bottom panels.

To account for the periodic nanoholes embedded in the multilayered structure (20 nm of a Au layer and 60 nm of SiO_2_), we implemented the dispersion relation between the wavevector |*k⃗*_spp_| and the reciprocal lattice vectors to illustrate that the interaction between an incident optical field and SPP obeys the conservation of energy and momentum (see Supporting Information for details). Figure 1a illustrates the reciprocal lattice vectors *G⃗*_*i,j*_ for the stacked hole arrays, the external SPP, gap SPP, and the light-line of the dielectric media. The relation between the SPPs and the conservation of momentum is linked to the reflectance resonance dip, as shown in Figure 1b.

Once the dispersion of the stacked layers is identified, the effect of the rectangular hole size on the resonance was investigated by a finite difference time domain (FDTD) solver (Ansys Lumerical FDTD Solutions). The reflection spectra of nanoholes with different sizes reveal the gradual change in the resonance wavelength from 830 to 750 nm. The design is composed of eight alternative layers of SiO_2_ and Au with periodic hole arrays. The periodicity of the hole is 400 nm in both *x*- and *y*-axes. The hole size is fixed along the *x*-axis (230 nm) and changed from 190 to 270 nm along the *y*-axis with a step size of 20 nm. Figure 1b presents the blue shift in the reflection spectra for the *y*-polarized illumination, with the gradual size increase of the periodic nanohole arrays (see the Methods section for details).

We calculated the mode profile at 640 and 780 nm for the 230 nm nanohole case, which corresponds to external SPP and gap SPP, respectively. In Figure 1c, the top two panels show the *y* component of the electric (*E*) field in the *x*−*y* plane. The bottom panels show the *x* component of the magnetic (*H*) field; the cutting plane was in the middle of the unit cell with normalized color codes. The first mode at 640 nm indicates that the fields are propagating on the top layer of the metal and air interface, an indication of external SPP. On the other hand, in the other mode, the fields are localized inside the hole and dielectric between the metal layers at 780 nm. Thus, the mode profile at 780 nm is evidence of the gap SPPs, which can be controlled by changing the hole size. Figure S2 presents *Ey* for a multiple unit cell at λ = 640 and 780 nm.

**Figure 2:**
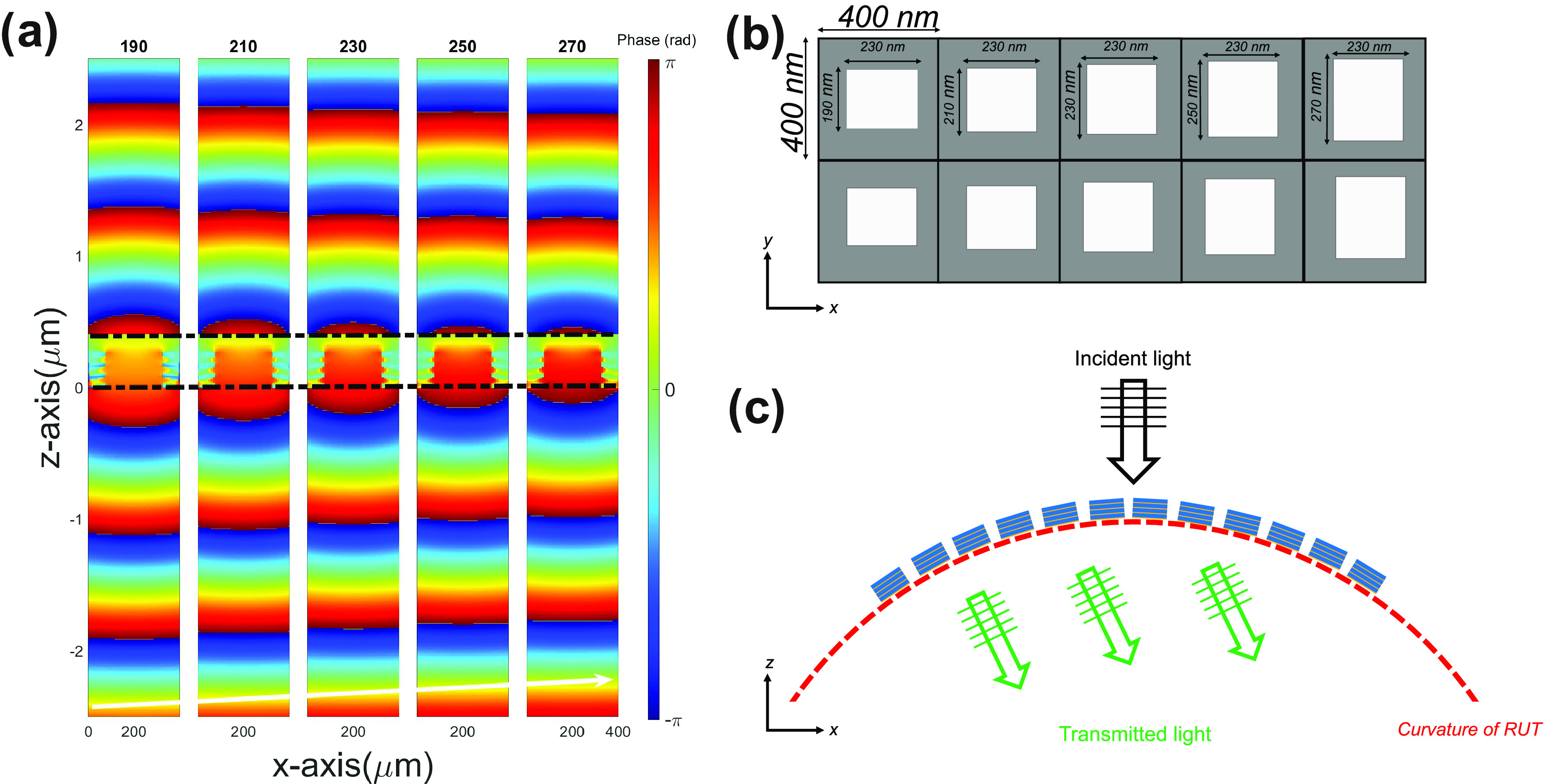
(a) Phase profile (units in radians) of the *y* component of the *E*-field up to 2λ from the nanoholes changing along the *y*-axis from 190 to 270 nm at λ = 750 nm. A white arrow is added for better visual comparison. (b) Top view of varied hole size structures is used to obtain wavefront control. A unit cell is composed of five different hole sizes. (c) Schematics of inclined wavefront transmitted inside the RUT with nanoholes on the top curvature.

As the modes provided by the different size nanoholes were identified, it is possible to bring them together in the design of the metasurface. To investigate this, the change in the phase of the *E*-field by increasing the hole size was simulated at λ = 750 nm. Figure 2a, shows the numerically calculated phase profile of five different hole sizes up to 2λ along the direction of the propagation (*z*-axis). As the hole size changes from 190 to 270 nm, the transmitted light experiences an additional phase. Thus, increasing the hole size from 190 to 270 nm gradually with a step size of 20 nm along the polarization direction (while keeping other parameters fixed) will introduce a gradual change of 0.95 radians in the phase. Although this can be optimized further for specific applications, this is sufficient to explore the proof-of-concept in this work.

The numerical results confirm that using a transition in the hole sizes leads to an inclined phase that can be used to control the wavefront for changing the propagation direction or focusing. Figure 2b presents the top view of the proposed design of the nanoholes with different side lengths changing from 190 to 270 nm along the *y*-axis, which is placed in a supercell of 2 μm. Although this simple planar design based on the metal-dielectric stacks works efficiently to control the wavefront, it is limited by the variation of the planar nanohole dimensions. Therefore, the additional phase control requires the extension of the metasurfaces design to out-of-plane. Motivated by the recent advances in the three-dimensional self-rolling RUTs and the fabrication of inverse metasurfaces, we combined these features in the out-of-plane metasurface on these 3D photonic microstructures. Figure 2c shows the cross-sectional view of the proposed metasurface design with the additional control, which is achieved by introducing a curve to the planar nanohole array.

## Results and Discussion

**Figure 3:**
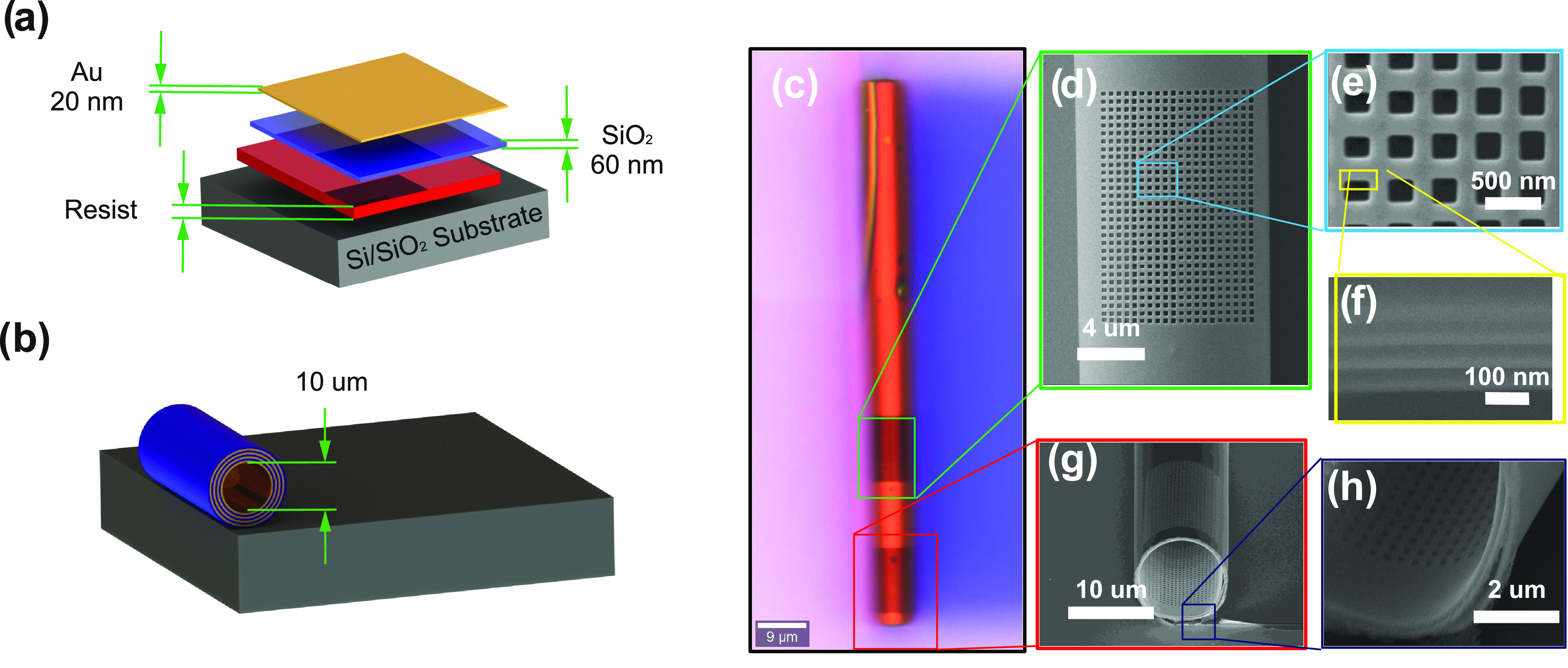
Sketch of the sample geometry showing the thin films deposited on top of the resist that rolls up from the substrate when resist was removed. (a) Thick resist (2 μm) was used as a sacrificial layer on the top Si/SiO_2_ substrate; 60 nm SiO_2_ and 20 nm Au were used as strained layers. (b) Released thin films resulting in the formation of RUT with a diameter of 10 μm. (c) Optical microscopy image of RUT with nanoholes. The scanning electron microscopy (SEM) images from (d, e) top, (f) cross section, and (g, h) bottom corner.

To experimentally realize out-of-plane metasurfaces for wavefront control, RUTs were fabricated using a self-rolling mechanism. The first rolled-up tubes were fabricated by Prinz et al. using a strained indium arsenide/gallium arsenide (InAs/GaAs) bilayer with lattice mismatch.^27^ Although the main fabrication approach is similar, we have adopted this technique to our study by changing the material layers and most importantly the sacrificial layer. The defined pattern was used to control the size and number of turns in the RUTs. This area was coated with 60 nm SiO_2_ and 20 nm Au. As these thin films inherit the strain due to the different deposition rates, this strain generates the RUT formation when released by etching/removing the sacrificial layer with acetone (see the Methods section for details). The diameter of the tubes was controlled by changing the thickness of SiO_2_ and Au. Additionally, the type of stress on each layer defines the direction of rolling.^37^ To achieve the rolling in an upward direction, we first deposit SiO_2_ with compressive stress at a low deposition rate and then Au with tensile stress by adding an adhesive layer of titanium (Ti) at a high deposition rate, as schematically shown in Figure 3a, b.

In addition to the thin-film stress, the type of sacrificial layer plays an important role in this process. The speed of rolling depends on the etch rate of the sacrificial layer, which also defines the diameter of the tube. The faster the sacrificial layer is removed, the more strained material is available to roll. Therefore, the size of the circumference of the tube gets bigger if the sacrificial layer is removed abruptly. For this study, a photolithography resist was chosen as a sacrificial layer. The resist was removed in 15 seconds (s), restricting the Au and SiO_2_ layers to get compact rolling compared to other sacrificial layers that take hours to etch. Therefore, the use of the resist not only yielded to bigger diameter microtubes compared to other sacrificial layers such as aluminum arsenide (AlAs)^27^ or germanium (Ge)^38^ but also provided a quick process with tight rolled layers.

We targeted a diameter of 10 μm with this process to introduce the effect of the curvature on the spectral response of the metasurfaces and also to overcome the characterization limitations. A tube with a diameter of 10 μm supplies a surface area that is big enough to accommodate 20× unit cells of 400 nm (or four supercells). In addition, the curvature of such diameter provides the out-of-plane effect with respect to the operating wavelength. Once the desired diameter of 10 μm is achieved, we specified the number of turns and the size of the tube by designing the rectangular pattern. The short side of the rectangle defines the length of the tube and the number of turns is the function of the longer side of the tube, as the rolling happens along the longer edge of the rectangle. A 75 × 125 μm^2^ rectangular pattern was used to achieve the 75 μm long tube with four turns, meaning eight alternative layers of SiO_2_ and Au. The nanohole arrays were obtained using focused ion beam (FIB) milling on the upper curvature of RUT. The optical microscopy image and the scanning electron microscopy (SEM) images of the milled area (8 × 12 μm^2^) (see the Methods section for details) are presented in Figure 3c–h. The quality of the tube is first confirmed using an optical microscope, as shown in Figure 3c. The FIB milling is performed on the center of the curvature to achieve better quality metasurfaces, presented in Figure 3d, as a complete device, and a supercell in Figure 3e. The cross-sectional SEM image is taken to confirm the number of bilayers, as shown in Figure 3f. The tilted images are used to measure the diameter and rolling quality of the tubes, as shown in Figure 3g, h, respectively.

**Figure 4:**
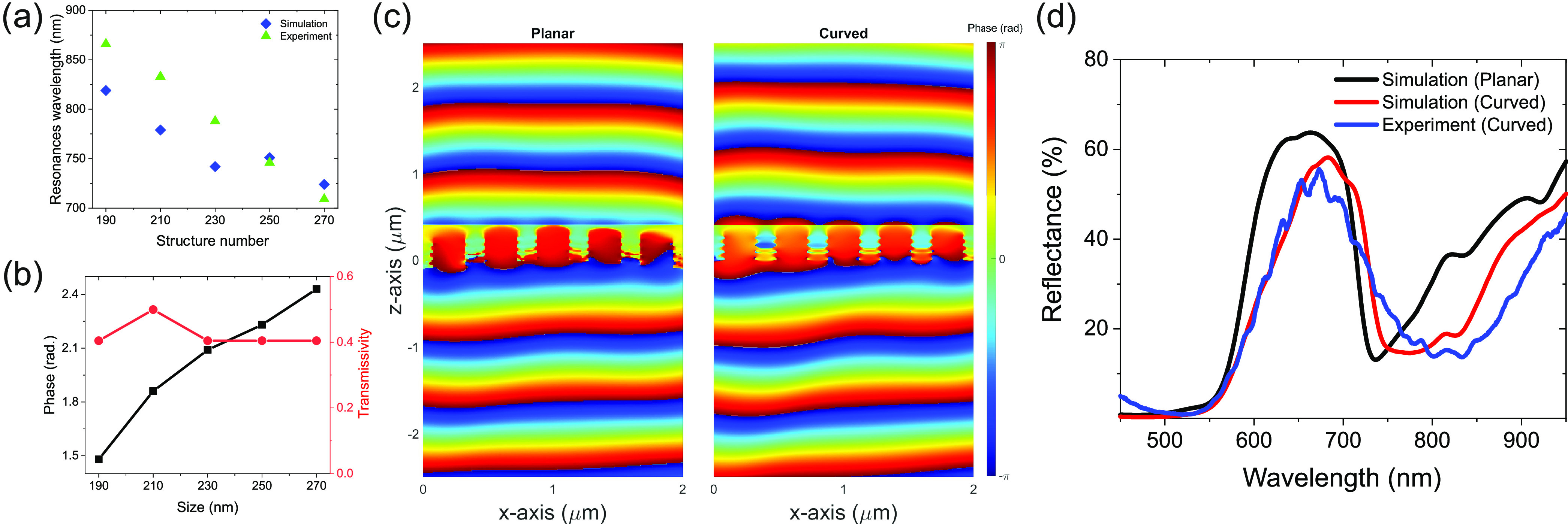
Comparison of different hole size (a) resonance dips in reflection, (b) transmission intensity and phase at λ = 750 nm, (c) phase response of the *y* component *E*-field at λ = 750 nm for planar and curved metasurfaces with different hole sizes, respectively, (d) simulated and measured reflectance spectra of the fabricated sample with nanoholes of different sizes from the planar (black) and curved metasurface (red (simulation) and blue (experiment)) along *y* polarization.

Additionally, metasurfaces formed by array of nanoholes with a constant size were fabricated for five different sizes on the same RUT. Figure 4a highlights the resonance value for five different hole sizes used in the design of the out-of-plane metasurfaces. The resonance from simulation (blue diamonds) and fabricated samples (green triangles) shows quite a good agreement. To control the wavefront of light, it is important to achieve a maximum phase difference. However, this phase difference should be achieved with low modulation in the intensity of the transmitted light. Figure 4b presents the comparison between the transmission intensity (red line with circles) and phase (black line with rectangles) of the transmitted light at λ = 750 nm, showing that the proposed metasurface has minimum intensity modulation. However, changing the hole size from 190 to 270 nm results in a ∼1 radian phase difference, which is suitable for the proof-of-concept phase control in out-of-plane metasurfaces.

Figure S3 comparatively presents the phase profile for two different cases (planar metasurfaces and curved metasurfaces) with a single hole size (190 nm). The planar metasurfaces with equal hole size confirm no phase gradient and the electromagnetic field propagates forward without any change in the propagation direction. Contrary to that, the curved metasurfaces with the same hole size slightly bend the light toward the center of the tube. This unique nature of the curved metasurface introduces an additional control to the light propagating inside the tube compared to that of the planar case. The curvature of RUT brings a new degree of freedom to control light in a more effective manner compared to planar metasurfaces.

Finally, we investigated the role of the curvature on the wavefront propagation inside the RUT with nanohole arrays. To identify this, first, the phase response from the metasurfaces with a uniform hole size at λ = 750 nm was calculated. Then, to mimic the effect of the curvature, we calculated the phase response of the metasurfaces with five different hole sizes in a supercell (2 μm). The change in the hole size along the *y*-axis introduces a gradual change in the phase, leading to steady wavefront control in one direction (Figure 4c). The change in the phase for the planar case is due to the change in the hole size. However, the phase response for the out-of-plane metasurfaces has additional bending, which is the outcome of the curvature of the tube. The curvature of the RUT defines the additional phase component for the out-of-plane nanohole-based metasurfaces. For our RUT, each hole has a tilt of 6° compared to the adjacent nanohole. We calculate the phase response of the 230 nm hole case for five different angles to see the effect of the curvature of the tube in addition to the hole size. Figure S4 presents the phase response of five different angles. The results show that the curvature of the RUT brings an additional phase control compared to the planar case, which only depends on the size of the hole. For a single unit cell, we obtained a change of 0.77 radian in the phase, while changing the tilt angle from 12° to −12° (see the Supporting Information for details).

The fabricated samples were characterized using a confocal microscope with results presented in Figure 4d. The simulation results are presented in the same figure for two different metasurfaces with planar (in black) and curved (in red) metasurfaces, which are illuminated by a *y*-polarized light source. The measurement (in blue) has a better agreement with the numerically calculated results for the curved metasurface design (see the Methods section for details). The spectral response of the supercell is an average of all of the individual holes. The experimental results show that the out-of-plane metasurfaces will be different from the planar metasurfaces. This new degree of freedom will allow better control of light and will open an entirely new avenue to utilize the light inside the tube. This controlled propagation of light can be used to selectively manipulate and sense the particles inside the RUT.

## Conclusions and Outlook

In conclusion, we introduced a new characteristic to the metasurfaces that use the advantage of a self-rolling mechanism to reduce the fabrication steps of multilayered structures and effectively control the optical field in contrast to planar metasurfaces. The dispersion relation for the metal/dielectric multilayer structures was analytically calculated to predict the resonance values for internal and external SPPs. In addition, we numerically calculated the resonance wavelength and the phase profile of each nanohole to design the metasurface. We experimentally realized the unique metasurfaces via the self-rolling mechanism and the FIB technique and measured the reflectance spectra of the fabricated metasurfaces. The numerically studied wavefront demonstrates the effect of the curvature on the wavefront defined by the metasurface. Another key result obtained in this study is the usage of the resist as a sacrificial layer. This yields a cost-effective, fast, and easy method to obtain RUTs that can be utilized in different metasurface-based applications.

While our samples demonstrate desired properties, it is possible to further improve the properties using the optimized RUT curvature for the operating wavelength. Additionally, the out-of-plane metasurfaces can be improved further using the neural network techniques for the metasurface design, better modeling approach,^39^ and increasing the number of meta-atoms (nanoholes) in a supercell. This will allow higher phase difference and even better wavefront control. Moreover, the signal-to-noise ratio/efficiency of meta-atoms can be improved using thin metal layers. Apart from that, our approach can be used to fabricate more complex structures on RUTs, for example, Babinet-inverted nanoantennas,^15^ to achieve metalenses for focusing and imaging purposes inside the tube. Overall, the results of this study open a unique platform for out-of-plane metasurface design, which provides possibilities for new kinds of metalenses, beam steering, and optical trapping of particles inside the RUTs. These applications may also lead to different platforms when integrated with already demonstrated properties of the RUTs such as wireless energy transfer, tunable shape, and neural guidance.

## Methods

### Numerical Simulations

The optical response of fabricated nanoholes inside the eight alternative layers of Au/SiO_2_ is numerically calculated using Ansys Lumerical FDTD Solutions. 3D electromagnetic simulations of reflection, phase, and *E*-field profiles were performed. We used the experimental dielectric functions provided in the literature to model Au^40^ and SiO_2_.^41^^41^ To obtain the optical response of nanoholes with different side lengths, we set our boundary conditions to periodic along the *x*- and *y*-axes and perfectly matched layer (PML) in the direction of propagation (*z*). The unit cell of size 400 nm was illuminated by a linearly polarized plane wave source in the *y* direction with a 450–950 nm spectral range. A conformal mesh of 4 nm was used in the simulation region while a finer mesh of 2 nm was employed in the region enclosing the multilayers to get better resolution.

The reflection results for a single unit cell with five different hole sizes were calculated along *y* polarization. However, the electric field was calculated by exciting the samples with the same linearly polarized total-field scattered-field (TFSF) source in the *y* direction with PML boundary conditions along *x*, *y*, and *z* axes. The complete supercell with the same and different holes was designed to see the effect of planar and curved RUTs on the wavefront control and electromagnetic field manipulations. A uniform mesh of 4 nm was used to obtain a better resolution in the distribution of the electric field.

### Fabrication

For the fabrication of RUTs, a 500 μm thick silicon (Si) substrate coated with 280 nm SiO_2_ was cleaned in acetone and isopropanol (IPA) with 10 minutes (min) sonication, and blow-dried under nitrogen (N_2_) flow. To clean the organic contaminants, oxygen plasma cleaning was done for 10 min. Once the samples were thoroughly cleaned, they were coated with a thin layer of hexamethyldisilazane (HMDS) at 125 °C to improve the adhesion of the photolithography resist. The AZECI3012 resist was spin-coated at 3000 rounds per minute (RPM) for 40 s. The resist was soft baked at 90 °C for 90 s. The spin-coated samples were exposed using the Suss MA6 mask aligner for 4 s under an ultraviolet (UV) lamp using the rectangular pattern in the mask. The postbaking process was done at 110 °C for 60 s. The samples were developed for 60 s using an MIF 726 developer and then rinsed three times in deionized water.

The developed samples were coated with 60 nm SiO_2_ using electron beam deposition at a rate of 0.1 nm/s. The thickness of SiO_2_ was confirmed using an ellipsometer. In the second step of deposition, the samples were coated with 2 nm Ti and 20 nm Au at 0.3 nm/s. The Ti layer was used for better adhesion of Au. Acetone was used to lift off the thin films on top of the resist. The samples were placed gently inside a beaker containing acetone. The samples were kept in acetone for 5 min, then moved to IPA for 30 s, and blow-dried under N_2_ flow. While removing the resist, SiO_2_ and Au layers started to roll due to the stress introduced during the deposition process. An optical microscope was used to ensure the quality of the tubes.

A Zeiss Crossbeam 540 FIB machine was used to mill the samples. The acceleration energy of Gallium ions was 30 KeV. The ion current used in the experiments was not determined because it was not calibrated, but a 1 pA probe was used.

### Optical Characterization

Reflectance spectra were measured using a Confocal Raman microscope from WiTec (alpha300R). The samples were excited with a broad-band light source (Energetiq EQ-99XFC LDLC, spectrum 190–2100 nm). The optical beam was focused on the curvature of the tube with metasurfaces using a Zeiss “Epiplan-Neofluar” 100× objective (NA = 0.7 WD = 0.31 mm) with a linear polarizer operating in the visible range. To detect the reflected signal, the same objective was used to collect the light in the normal direction. The collected light was coupled to an optical fiber connected to an Ocean Optics Flame UV–vis Spectrometer, with a detection range from 400 to 980 nm. We first measured the reflection spectrum from a reference Ag mirror. Then, we measured the reflection spectrum of the nanorectangle array on the curved RUT. The reflectance spectra (reflectance) were calculated according to the following formula
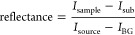
1where *I*_sample_ is the collected reflection spectrum of the nanostructures; *I*_source_ is the acquired spectrum of the broad-band light source, which is measured using a perfect reflector; *I*_sub_ is the substrate reflection, acquired from the unpatterned area of the Si/SiO_2_ substrate; and *I*_BG_ stands for the background counts, acquired by the used system.

## Supporting Information Available

The video file showing the formation of the tubes (MP4)
The dispersion relation of the multilayered metamaterial and the phase of *E*-field response of the supercell with the same hole sizes for planar and curved cases (PDF)
