# Supplementary material for: Wavefront Control with Nanohole Array-Based Out-of-Plane Metasurfaces
Source: ACS Appl Nano Mater. 2021 Aug 2;4(9):8699–705. doi: 10.1021/acsanm.1c01178 (PMC8477370; doi:10.1021/acsanm.1c01178)
Supplement: Supplementary file 2 — an1c01178_si_002.pdf [file an1c01178_si_002.pdf]

Supporting Information: Wavefront Control with Nanohole Array-based Out-of-plane Metasurfaces

Mohsin Habib, Ibrahim Issah, Daria Briukhanova, Alireza R. Rashed, and Humeyra Caglayan*

Faculty of Engineering and Natural Science, Photonics, Tampere University, 33720 Tampere, Finland

E-mail: humeyra.caglayan@tuni.fi

Dispersion relation calculations

The dispersion relation of multilayered metamaterial helps to determine the existence of SPPs excited through a metal layer sandwiched between two dielectric media. To comprehend the excitation of internal and external SPPs in stacked hole RUTs, the dispersion relation is formulated using the Helmholtz equations to determine the field solutions along the propagation direction as well as the evanescent fields confined in the transverse direction of the multilayered structure. As shown in Figure S1 (a), the layers were assumed to be parallel in the x-axis with a relative permittivity dependent on one spatial coordinate ($\epsilon = \epsilon(z)$). The y-direction of the multilayered structure is considered to be infinite and homogeneous ($\partial/\partial y = 0$). The transverse confined field in the z-direction is related to the attenuation coefficients in two metallic media with a field dependence of $z > t_d/2 + t_m$, $t_d/2 < z < (t_d/2 + t_m)$, $-t_d/2 < z < t_d/2$, and $(-t_d/2 + t_m) < z < -t_d/2$ as shown in Figure S1 (a). Here we considered the transverse magnetic (TM) plasmonic modes in each region by applying the required boundary conditions. We defined the vector potentials of

the electric field and magnetic field as $\vec{F}$ and $\vec{A}$, respectively.

For simplicity, we limit the formulation to the symmetric case whereby both the semi-infinite layers relative to the dielectric constants are equal to $\epsilon_1 = \epsilon_2 = \epsilon_{air}$ while $\epsilon_{m1} = \epsilon_{m2} = \epsilon_{Au}$ depicts the Au region. ϵ_d represents the permittivity of the sandwiched dielectric region. Based on the aforementioned conditions, the $\vec{A}$ can be expressed as $A_z\hat{z} = Ae^{k_z z}e^{i\beta x \hat{z}}$, where $e^{k_z z}$ describes the electromagnetic field depth dependence with $k_z = \pm\sqrt{\beta^2 - k_0^2\epsilon}$ and $k_0 = 2\pi/\lambda$. k_0 depicts the vacuum wavenumber and propagation wavenumber along x direction shown in Figure S1 (b) is represented as β. Since metals are lossy, the field penetration depth is minimal and essentially the dominant field will be surface waves concentrated at the metal-dielectric interfaces. The TM modes are obtained by solving the electromagnetic field components using Maxwell's equations which are represented as follows:

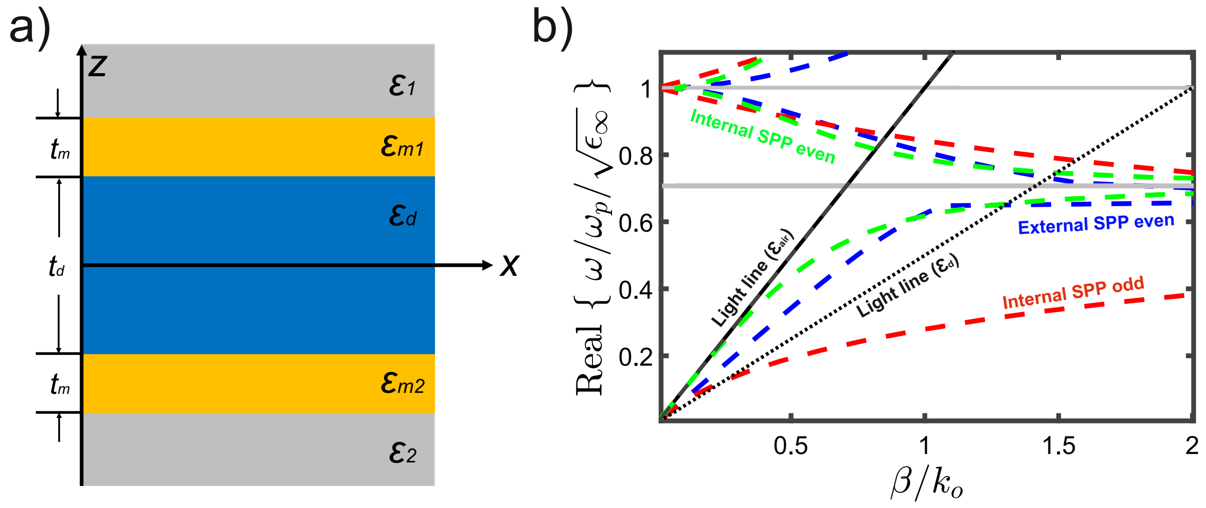

Figure S1: (a) Geometry of a metal/dielectric interface, with a dielectric sandwiched between two metal layers. (b) Dispersion relation of SPPs excited by the metal-insulator-metal structure that depicts the internal and external SPP modes which propagate along the internal and external metal-insulator interfaces. The light line ϵ_{air} and ϵ_d show the dispersion relation in free space and the dielectric medium, respectively. The SPPs even and odd show the symmetric and anti-symmetric modes excited by a dielectric sandwiched between two metal layers.

$$E_x = -j\frac{1}{\sigma\mu\varepsilon}\frac{\partial^2 A_z}{\partial x \partial z}, \quad H_x = 0 \tag{S1}$$

$$E_y = 0, \quad H_y = -\frac{1}{\mu}\frac{\partial A_z}{\partial x} \tag{S2}$$

$$E_z = -j\frac{1}{\varpi\mu\varepsilon}\left(\frac{\partial^2}{\partial z^2} + k_0^2\varepsilon\right)A_z, \quad H_z = 0. \tag{S3}$$

Solving equation set S1-S3 and applying the boundary conditions yields the field solutions which are represented below;

$$\begin{aligned} &\text{for } z > t_d/2 + t_m \\ &H_y = -j\tfrac{\beta}{\mu}Be^{-k_2 z}e^{j\beta x} - j\tfrac{\beta}{\mu}Ce^{k_2 z}e^{j\beta x} \\ &E_x = -\tfrac{\beta k_2}{\varpi\mu\varepsilon_0\varepsilon_{m1}}Be^{-k_2 z}e^{j\beta x} + \tfrac{\beta k_2}{\varpi\mu\varepsilon_0\varepsilon_{m1}}Ce^{k_2 z}e^{j\beta x} \\ &E_z = -j\tfrac{\beta^2}{\varpi\mu\varepsilon_0\varepsilon_{m1}}Be^{-k_2 z}e^{j\beta x} - j\tfrac{\beta^2}{\varpi\mu\varepsilon_0\varepsilon_{m1}}Ce^{k_2 z}e^{j\beta x} \end{aligned} \tag{S4}$$

$$\begin{aligned} &\text{for } t_d/2 < z < (t_d/2 + t_m) \\ &H_y = -j\frac{\beta}{\mu}De^{-k_3 z}e^{j\beta x} - j\frac{\beta}{\mu}Ee^{k_3 z}e^{j\beta x} \\ &E_x = -\tfrac{\beta k_3}{\varpi\mu\varepsilon_0\varepsilon_d}De^{-k_3 z}e^{j\beta x} + \tfrac{\beta k_3}{\varpi\mu\varepsilon_0\varepsilon_d}Ee^{k_3 z}e^{j\beta x}, \\ &E_z = -j\tfrac{\beta^2}{\varpi\mu\varepsilon_0\varepsilon_d}De^{-k_3 z}e^{j\beta x} - j\tfrac{\beta^2}{\varpi\mu\varepsilon_0\varepsilon_d}Ee^{k_3 z}e^{j\beta x} \end{aligned} \tag{S5}$$

$$\begin{aligned} &\text{for } -t_d/2 < z < t_d/2 \\ &H_y = -j\tfrac{\beta}{\mu}Fe^{-k_4 z}e^{j\beta x} - j\tfrac{\beta}{\mu}Ge^{k_4 z}e^{j\beta x} \\ &E_x = -\tfrac{\beta k_4}{\varpi\mu\varepsilon_0\varepsilon_{m2}}Fe^{-k_4 z}e^{j\beta x} + \tfrac{\beta k_4}{\varpi\mu\varepsilon_0\varepsilon_{m2}}Ge^{k_4 z}e^{j\beta x}, \\ &E_z = -j\tfrac{\beta^2}{\varpi\mu\varepsilon_0\varepsilon_{m2}}Fe^{-k_4 z}e^{j\beta x} - j\tfrac{\beta^2}{\varpi\mu\varepsilon_0\varepsilon_{m2}}Ge^{k_4 z}e^{j\beta x} \end{aligned} \tag{S6}$$

$$\begin{aligned} &\text{for } (-t_d/2 + t_m) < z < -t_d/2 \\ &H_y = -j\tfrac{\beta}{\mu}He^{k_5 z}e^{j\beta x} \\ &E_x = \tfrac{\beta k_5}{\varpi\mu\varepsilon_0\varepsilon_2}He^{k_5 z}e^{j\beta x} \\ &E_z = -j\tfrac{\beta^2}{\varpi\mu\varepsilon_0\varepsilon_2}He^{k_5 z}e^{j\beta x}, \end{aligned} \tag{S7}$$

where ϖ, μ, and ε_0 represent the angular frequency, permeability, and relative permittivity of the multilayered media, respectively. From the above equations, the dispersion relation

can be obtained by solving the system of linear equations and applying continuity boundary conditions at every metal-dielectric interface expressed as,

$$-\frac{\left(\frac{k_4}{\varepsilon_{m2}}-\frac{k_3}{\varepsilon_{t_d}}\right)\left(\frac{k_5}{\varepsilon_2}-\frac{k_4}{\varepsilon_{m2}}\right)e^{-k_4t_m}e^{k_3t_d/2}+\left(\frac{k_4}{\varepsilon_{m2}}+\frac{k_3}{\varepsilon_{t_d}}\right)\left(\frac{k_4}{\varepsilon_{m2}}+\frac{k_5}{\varepsilon_2}\right)e^{k_4t_m}e^{k_3t_d/2}}{\left(\frac{k_4}{\varepsilon_{m2}}+\frac{k_3}{\varepsilon_{t_d}}\right)\left(\frac{k_5}{\varepsilon_2}-\frac{k_4}{\varepsilon_{m2}}\right)e^{-k_4t_m}e^{-k_3t_d/2}+\left(\frac{k_4}{\varepsilon_{m2}}-\frac{k_3}{\varepsilon_{t_d}}\right)\left(\frac{k_4}{\varepsilon_{m1}}+\frac{k_5}{\varepsilon_2}\right)e^{k_4t_m}e^{-k_3t_d/2}}=0 \quad \text{(S8)}$$

and simplified to

$$\left(\frac{k_4}{\varepsilon_{m2}}+\frac{k_3}{\varepsilon_{t_d}}\right)\left(\frac{k_4}{\varepsilon_{m2}}+\frac{k_5}{\varepsilon_2}\right)e^{k_4t_m}e^{k_3t_d/2}=\left(\frac{k_4}{\varepsilon_{m2}}-\frac{k_3}{\varepsilon_{t_d}}\right)\left(\frac{k_5}{\varepsilon_2}-\frac{k_4}{\varepsilon_{m2}}\right)e^{-k_4t_m}e^{k_3t_d/2}, \quad \text{(S9)}$$

and describes the SPPs in the multilayered structure. The dispersion relation helps to predict analytically the extraordinary optical transmission (EOT) peaks from the excitation of both internal and external SPP modes. This model formulated to depict the EOT peaks at different spectral wavelengths does not consider the holes stacked in the multilayered structure. Stacked holes can be accounted for by implementing the conservation of momentum and energy between the incident optical field and the periodicity of the rectangular array. The relation of SPPs and conservation of momentum can be expressed as

$$\left|\vec{k}_{spp}\right|=\left|\vec{k}_x+\vec{G}_{i,j}\right|=\left|\vec{k}_0\sin\phi+i\vec{G}_x+j\vec{G}_y\right| \quad \text{(S10)}$$

where $\left|\vec{k}_{spp}\right| \equiv \beta(\varpi)$ is the wave vector of the SPP, $\left|\vec{G}_x\right| = 2\pi/a_x$, and $\left|\vec{G}_y\right| = 2\pi/a_y$ are the reciprocal lattice vectors for the stacked holes, and $\vec{k}_0\sin\phi$ is the in-plane component of the incident wave vector. a_x and a_y are the lattice periodicity in the x and y-directions. Notably, the normalized frequency at large Bloch wave vectors tends to approach the SPP frequency, which is expressed as

$$\varpi_{sp}=\frac{\varpi_p}{\sqrt{\varepsilon_\infty+\varepsilon_i}} \quad \text{(S11)}$$

with $\varepsilon_i = \varepsilon_{air}$ or ε_d for external or internal SPPs, respectively. However, for a short wave

vectors $\beta \ll k_p$, the SPP propagation constant is related to the internal SPPs, and is expressed as

$$\beta^{\text{int}} \equiv \left|\vec{k}_{sp}^{\text{int}}\right| = \left|\vec{k}_o\right| \sqrt{\varepsilon_{t_d}} \left[\frac{t_d}{t_d + \lambda_p \coth\left(k_p t_m\right)/\pi}\right]^{-1/2}, \tag{S12}$$

where $k_p = 2\pi/\lambda_p$, and the external frequency of the low frequency range can be formulated as

$$\beta^{\text{ext}} \equiv \left|\vec{k}_{sp}^{\text{ext}}\right| = \left|\vec{k}_o\right| \sqrt{\frac{\varepsilon_{\text{air}}\varepsilon_m}{\varepsilon_{\text{air}} + \varepsilon_m}}. \tag{S13}$$

E-field response

Figure S2 presents the E-field response of multiple unit cells at 640 and 780 nm.

Phase response of uniform hole size

The phase of the E-field response of the supercell with the same hole sizes for planar and curved cases are presented in Figure S3. Figure S4 shows the E-field for a single hole with different tilt angles.

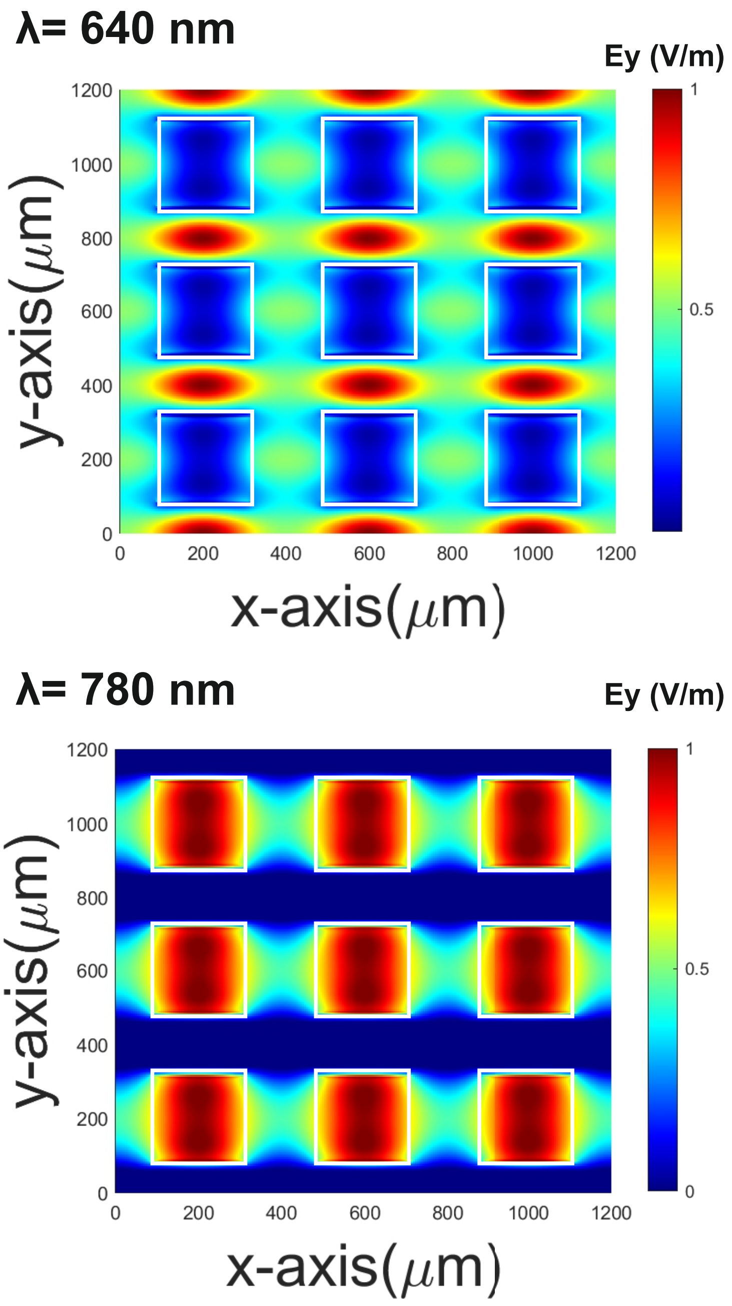

Figure S2: The y component of E-field for 3x3 nanohole array at 640 and 780 nm.

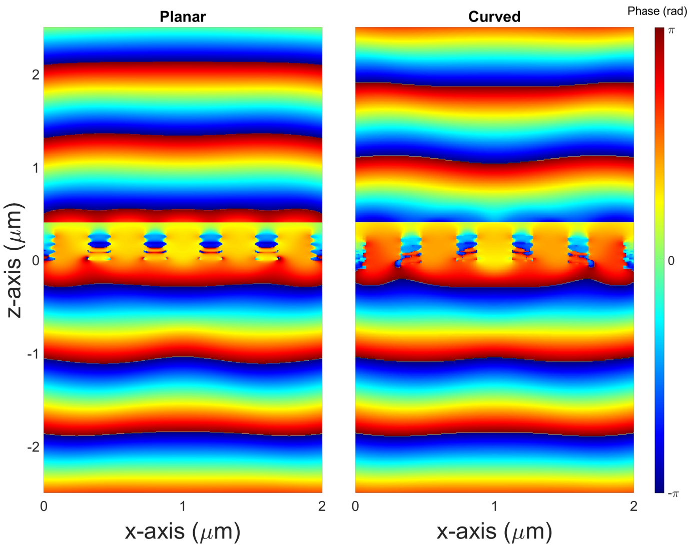

Figure S3: Phase response of the y-component of the E-field at λ= 750 nm for planar and curved metasurfaces with same hole size, respectively.

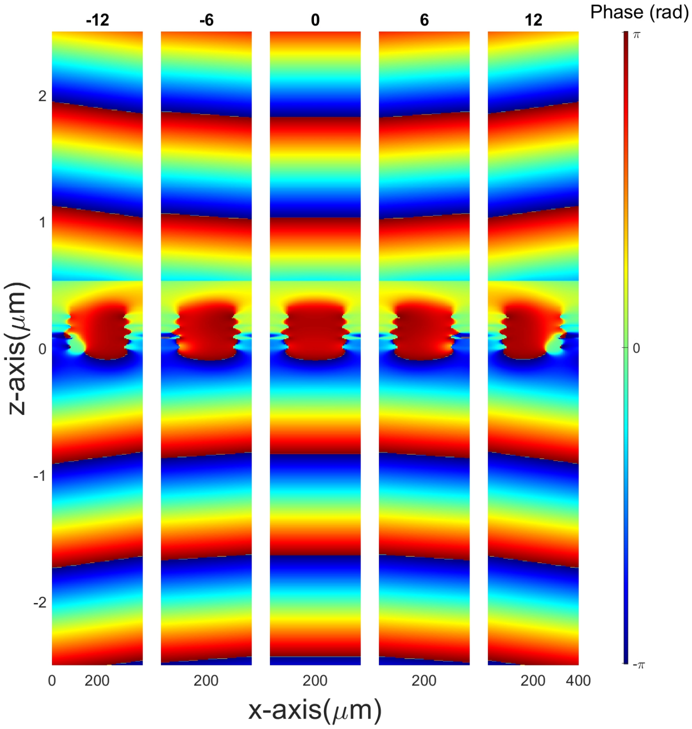

Figure S4: Phase response of the y-component of the E-field at λ= 750 nm for five different tilt angles -12°, -6°, 0°, 6° and 12°.
